# A novel killed oil adjuvanted bovine viral diarrhea virus vaccine protects from viremia and clinical manifestations: an immune response and challenge study in cattle

**DOI:** 10.1007/s11259-025-10792-y

**Published:** 2025-06-13

**Authors:** Berfin Ertürk, Gizem Aytoğu, Kadir Yeşilbağ

**Affiliations:** 1 Department of Virology, Faculty of Veterinary Medicine, Dicle University, Diyarbakır, 21200 Turkey; 2 Department of Virology, Faculty of Veterinary Medicine, Bursa Uludag University, Bursa, 16059 Turkey

**Keywords:** Bovine viral diarrhea virus, Killed vaccine, Local strains, Challenge study, Neutralising antibody

## Abstract

Bovine viral diarrhea virus continues to threaten animal health with serious economic losses worldwide. Various killed, live-modified, or recombinant vaccine strategies are being developed for protection and control against this virus. The most important thing discovered is the choice of local and widespread strains in the vaccine content. In this study, the effectiveness of a Montanide^®^ ISA 206 adjuvanted killed trivalent vaccine containing endemic local strains (TR-21 [BVDV-1*l*], TR-26 [BVDV-1f], and TR-15 [BVDV-2b]) isolated from Türkiye, was evaluated with a cattle challenge study. Experimental groups were designed as single dose vaccination (Group-I, n:11), two dose vaccination (Group-II, n:11), and unvaccinated (Group-III, n:6) with male calves aged about 6 months. Following the immunization, challenge virus (TR-72 [BVDV-1*l*], TCID_50_ 10^6.5^) was given intranasally to each group (5 animals in Group-I and II and 4 animals in Group-III), and clinical findings, hematological changes, virus shedding, side effects, and viremia were monitored for 14 days after inoculation. Serological monitoring of the remaining animals against homologous and heterologous strains was carried out at one-month intervals between the 21st and 201st days after the first vaccination. The obtained results showed that the viremia, hematological changes, and clinical findings shown in unvaccinated animals (Group-III) were significantly suppressed (*p* < 0.05) in the vaccinated groups (Group-I and II). In addition, it was found that serologically monitored animals maintained protective neutralizing antibody (nAb) titers ≥ 8 log_2_ for vaccinal and also for all reference BVDV-1 strains, which is more than three-fold protective antibody response, lasting more than 7 months after the first vaccination, whether in single or two dose application. The rise of nAbs was also detected for heterogous BVDV strains. The detected nAb titers were significantly higher (*p* < 0.05) in the two dose vaccination group. Based on the results, it was concluded that this trivalent- inactivated vaccine candidate can protect cattle against acute BVDV infections.

## Introduction

Bovine viral diarrhea virus (BVDV), belongs to the *Pestivirus* genus of the *Flaviviridae*, is one of the most important pathogens of livestock and causes serious economic losses worldwide. BVDV is divided into *Pestivirus A* (BVDV-1), *Pestivirus B* (BVDV-2), and *Pestivirus H* (BVDV-3) based on genetic and antigenic properties. Just recently, Pestivirus A, Pestivirus B, and Pestivirus H have been updated as *Pestivirus bovis*, *Pestivirus tauri*, and *Pestivirus braziliense*, respectively, by the regulation of the International Committee on Virus Taxonomy (Murilo et al. 2023). Pestivirus subgenotypes have been defined according to the 5’UTR or the Npro coding regions, which are frequently used in taxonomic and epidemiological studies, resulting in 24 *Pestivirus bovis* subgenotypes (BVDV-1a to −1x) and 5 *Pestivirus tauri* subgenotypes (BVDV-2a to −2e) (Yeşilbağ et al. 2017; Fritzen et al. 2024). There are also two biotypes of the virus, cytopathogenic (cp) and non-cytopathogenic (ncp), in cultured cells (de Oliveira et al. 2022).

Depending on the gestational period and viral ncp biotype, BVDV infection prenatally results in early embryonic death, teratogenic effects on the fetus, and the development of persistently infected animals in the first trimester of gestation. Persistently infected (PI) animals carry and share the virus for a lifetime, while acutely infected animals shed the virus shorthly; thus, both pose a risk to susceptible animals (Goto et al. 2021). In the postpartum period, BVDV causes acute infections with different clinical signs due to multi-tissue tropism, such as the reproductive system, central nervous system, mucosal epithelial tissue, bone marrow, peripheral blood, and lymphoid tissues (Goto et al. 2021). BVDV also causes depletion of circulating B- and T-lymphocytes, leading to immunosuppression that favors infections with other viral or bacterial pathogens. Moreover, a fatal form of mucosal manifestations (Mucosal disease, MD) develops when persistently infected calves are super-infected with the cp virus strain (Chang et al. 2021). In Europe, the total annual economic loss from BVD infection, known as the third most economically important disease, is approximately 17–150 million dollars per year (van Duijn et al. 2019).

The virus is exposed to a high risk of mutation during each replication cycle (Chang et al. 2021). The emergence of new strains and variants poses a significant constraint to the efficacy of commonly used vaccines. The eradication of BVDV mainly depends on eliminating PI animals, vaccination, and biosecurity (Fulton et al. 2020). The meticulous and continuous monitoring of the virus strains and variants, the development of the most up-to-date diagnostic methods with high efficiency to detect various strains and variants, and the development of effective homologous vaccines against the circulating virus are crucial (Ridpath et al. 2010). The commercial BVDV vaccines available in the market, produced by conventional technologies, are either killed or live-modified vaccines that may include either BVDV-1 (*Pestivirus bovis*) or BVDV-2 (*Pestivirus tauri*) viruses. The BVDV-1 is the dominating virus species (Yeşilbağ et al. 2017) at the global scale, and the used vaccinal strain is generally selected from the BVDV-1a subgroup (Fulton et al. 2020). Field experiences pointed out the possible insufficiencies of the BVDV vaccines containing common vaccine strains against local strains and subgenotypes antigenically different from BVDV-1a (Ridpath et al. 2010; Alpay and Yeşilbağ 2015; Sozzi et al. 2020).

This study aimed to investigate an inactivated oil adjuvanted BVDV vaccine containing a combination of three BVDV subgenotypes, which contain a predominant local strain, and the efficacy of a new vaccine prototype tested in a cattle challenge model.

## Materials and methods

### The animals, vaccine formulation and administration

Animals were selected from Holstein-Fresian breeds, ranges 6.0 to 6.9 month-old (mean 6.4, median 6.4) male calves. Before starting the experiment, animals were tested for freedom from BVDV infection by a commercial BVDV Ag ELISA kit (IDEXX, Switzerland). At the same time, a serum neutralization test against the BVDV-NADL strain was performed, and the animals free from both BVDV antigens and antibodies were included in the experiment. The selection of vaccine formulation has already been performed by preclinical studies, and the Montanide ISA 206 (Seppic, France) adjuvanted (1:1) BEI-inactivated trivalent vaccine, has previously been shown to be immunogenic and safe in experimental animals following the USA Code of Federal Regulation (USA-CFR 1987), was used (Kadiroğlu and Yeşilbağ 2025). BVDV-l*l* and BVDV-1f, which are the common subgenotypes in the field, were taken into account when choosing the strains for the vaccine preparation (Yeşilbağ et al. 2017). The vaccine contains 10^6^,10^5.6^, and 10^5.5^ TCID_50_ per dose of the each of ncp strains BVDV-1*l* (strain TR-21), BVDV-1f (strain TR-26), and BVDV-2b (strain TR-15) originally obtained from different geographical regions of Turkiye, respectively (Yeşilbağ et al. 2008). Selected 28 calves were divided into three experimental groups; Group I: single dose vaccination (11 animals), Group II: two dose (prime-booster) vaccination (11 animals), and Group III: control group (6 animals). Two milliliters of the vaccine were administered intramuscularly to each animal in the vaccination groups I and II. Experimental group I received only one dose of the vaccine (first vaccination) while a prime&booster vaccination with a 21-day interval (one dose, second vaccination) was administered to group II.

### Immunogenicity of the vaccine

Randomly selected 6 calves from each of the vaccinal groups (groups I and II) were separated for immunogenicity and follow-up studies. From these animals, blood samples were collected on the 21st, 51st, 81st, 111st, 141st, 171st, and 201st days after the first vaccine delivery. After heat inactivation at 56 °C, the resultant serum fraction was kept in a freezer at −20 °C until testing. Sera were evaluated for protective neutralizing antibody titers against homologous vaccine strains (BVDV TR-21, TR-26, and TR-15) or reference heterologous (NADL [BVDV-1a] and Gi-II [BVDV-2b]) strains. Briefly, inactivated sera were serially diluted twofold, and equal volumes of 100TCID_50_ test virus suspension were added to 96-well plates. After one hour of incubation at 37 °C, 5% CO_2_ atmosphere, MDBK cell suspension (2 × 10^5^ cells/ml) was distributed and plates were followed for at least 5 days. Evaluation of the test results for cytopathogenic strains (BVDV NADL and Gi-II) was performed by microscopic examination, while the immunoperoxidase monolayer assay (IPX) protocol was applied to evaluate the results for ncp strains TR-21, TR-26, and TR-15 (Alpay and Yeşi̇lbağ 2019). To perform the IPX assay, after dry heat fixation, 0.5% O-D-glucopyranoside (Sigma, St. Louis, MO, USA) solution was added and the plates were incubated at room temperature for 10 min. Following washing, monoclonal mouse antibody specific for the NS3 protein of pestiviruses (pool 1/4/7), biotin-labeled antimouse antibody (Pierce, Rockford, USA), and streptavidin-biotinylated-HRPO conjugates (Pierce, Rockford, USA) were added sequentially and incubated. The following substrate solution (2 mg AEC in 0.3 mL DMF, 4.7 mL Na-acetate buffer [pH 5.5], and 0.05% H_2_O_2_) were used. Antibody titers were calculated as the final dilution of duplicate wells in which no IPX staining or cytopathogenic effects were observed.

### Experimental challenge, clinical assessment, and sampling

On the 51st day after the start of the applied vaccination scheme, 5 animals from each of group I and group II, as well as 4 animals from the unvaccinated group (group III) were subjected to experimental challenge. The strain TR-72, belonging to the BVDV-1*l* subgenotype and isolated from a naturally infected animal in the Northwest region of Turkiye (Yeşilbaǧ et al. 2014), was used as the challenge virus. The animals were inoculated by intranasal instillation with the second passage levels of BVDV strain TR-72 (TCID_50_ 10^−6.5^) in a 10 ml medium as the challenge virus. Specimens were taken on days 2 through 14 after the challenge and animals were monitored for clinical findings such as fever, nasal discharge, lacrimation, hyperemia, cough, oral lesions, diarrhea, and possible changes in general conditions. Also, animals in all the groups were observed for possible side effects for 7 days after intramuscular vaccine administration. EDTA blood, serum samples, nasal and rectal swabs were daily collected from the animals for laboratory testing. The samples were used for virus isolation (VI), antigen ELISA, and real-time RT-PCR analyses. To prepare inoculum, rectal and nasal swab samples were immersed in 2 ml of PBS, then vortexed for 1 min, and the contents passed through a 0.22 nm injector filter. The buffy coating was recovered after centrifuging EDTA blood samples at 800 x g for 10 min. The cells were reconstituted with phosphate buffered saline (PBS) with 10% DMSO. Serum samples were obtained from clot activator tubes and then stored frozen at − 70 °C until tested. Analysis of blood counts were determined by an automated veterinary hematology system (Hasvet VH5R, Hasvet Medical, Turkiye). It was focused on the changes in the number of thrombocytes, white blood cells (WBC), and erythrocyte counts.

### Determination of viral antigens and viral load

The commercial antigen ELISA kit for BVDV E^rns^ antigens (IDEXX, Switzerland) was implemented to test all the blood, nasal, and rectal swab samples in the three study groups. The manufacturer-recommended test protocol was followed. The ELISA reader (Thermo Fisher Scientific’s Multiskan EX, Vantaa, Finland) was utilized to analyze the test at 450 nm.

In order to extract viral RNA from each PBMC sample, a commercial nucleic acid isolation kit (Macherey-Nagel Nucleospin Virus) was employed. The IDEXX RealPCR BVDV RNA Kit (IDEXX, Montpellier, France) was used for real-time RT-PCR. The reaction volume of 25 µL was composed of 10 µL BVDV RNA mix, 10 µL RNA MMx (master mix), 4.5 µL nuclease-free water, and 0.5 µL sample RNA. The BVDV-specific probes were 5′-end labeled with 6-carboxyfluorescein (FAM) and Hexachloro-Fluorescein (Hex) fluorescent dye, respectively. Real-time RT-PCR was performed using QIAGEN Rotor-Gene Q real-time PCR equipment under the following conditions: 50 °C for 15 min and 95 °C for 1 min followed by 45 cycles of 95 °C for 15 s and 60 °C for 30 s. Applying Q-Rex software version 2.3.5 for analyzing the real-time RT-PCR results, the genomic copies of each sample were assessed based on the Ct values. In the validated test protocol the samples having a Ct value less than 30 were accepted positive for the BVDV genome. Real-time RT-PCR assay was applied to Groups-II and III to compare the inhibition rate of viremia for the suggested two dose vaccination scheme to the unvaccinated group.

### Virus isolation

For virus isolation in a 24-well plate, the Madin-Darby bovine kidney (MDBK) cell cultures (1 × 10^5^ cells/ml) were seeded in each well of the plate. The cell culture was maintained in Dulbecco’s modified eagle medium (DMEM) containing 10% fetal bovine serum (FBS), 1% penicillin-streptomycin, and 5% CO_2_. After confluency, 200 µl of samples were inoculated onto separate wells, and after a short incubation at 37 °C for 1 h, the inoculated wells were rinsed and serum-free DMEM was distributed. Because of the ncp nature of the challenge strain (BVDV TR-72), the virus replicated in the plates was evaluated by IPX after 5 days post-incubation (Alpay and Yeşi̇lbağ 2019).

### Statistical analysis

The Mann-Whitney U test was used to analyze clinical data, ELISA, and real-time RT-PCR values. The Paired Sample T-test was applied to analyze antibody responses produced against different viruses at various times to assess the vaccine’s efficacy. All statistical data were obtained from IBM SPSS Statistics 26 (Chicago, IL, USA).

## Results

### Immunogenicity and duration of immunity

The mean neutralizing antibody titers of the vaccinated animals not included in the challenge study (*n* = 6) are shown in Table 1. In the single dose vaccination (group-I), nAb titers for homologous (vaccinal) strains were detected to be close to each other and were high (> log 6) after the 51st day of immunization. In addition, antibody titers against heterologous (reference) strains peaked at the 111st day or later. In the two dose vaccination (group-II), it was shown that the nAb titers for homologous strains were quickly increased starting at the earliest sampling date of the 21st day. Antibody titers of reference strains started to increase from the 51st day and remained high until the end of the experimental period in group-II. Mean neutralizing antibody titers ranged from 2.38 to 8.88 log_2_ in the group-I and 4.71–8.97 log_2_ in the group-II. When we compared the antibody response of single and two dose vaccinated animals against the strains, it was found that the two dose vaccination group was significantly higher against the BVDV-Gi-II strain (*p* < 0.05).

**Table 1:** Time-depended geometric mean and standard deviations of neutralizing antibody titers (log_2_) detected in the experimental vaccine groups against homolog and heterologous strains

		Days after immunization						
	Test Virus	21st	51st	81st	111st	141st	171st	201st
Group I (Single Dose Vaccination)	TR-21 (−1*l*)	8.33 ± 1.63^a^	9.00 ± 0.00^a^	9.00 ± 0.00^a^	9.00 ± 0.00^a^	9.00 ± 0.00^a^	9.00 ± 0.00^a^	8.83 ± 0.40^a^
	TR-26 (−1f)	2.33 ± 2.42^b^	6.80 ± 1.93^b^	6.33 ± 2.06^b^	8.00 ± 2.00^a^	9.00 ± 0.00^a^	8.83 ± 0.40^a^	7.83 ± 1.16^a^
	TR-15 (−2b)	5.33 ± 1.63^b^	8.20 ± 1.60^ab^	9.00 ± 0.00^a^	8.16 ± 2.04^a^	8.83 ± 0.40^a^	9.00 ± 0.00^a^	4.33 ± 0.51^b^
	NADL (−1a)	1.00 ± 0.00^c^	1.00 ± 0.00^c^	1.00 ± 0.00^c^	6.33 ± 3.01^a^	8.83 ± 0.40^a^	9.00 ± 0.00^a^	9.00 ± 0.00^a^
	Gi-II (−2b)	1.00 ± 0.00^c^	1.00 ± 0.00^c^	1.00 ± 0.00^c^	1.83 ± 1.60^b^	3.00 ± 0.89^b^	4.33 ± 1.21^b^	4.50 ± 1.04^b^
Group II (Two Dose Vaccination)	TR-21 (−1*l*)	9.00 ± 0.00^a^	9.00 ± 0.00^a^	9.00 ± 0.00^a^	9.00 ± 0.00^a^	9.00 ± 0.00^a^	9.00 ± 0.00^a^	8.85 ± 0.37^a^
	TR-26 (−1f)	5.00 ± 2.64^b^	7.20 ± 2.03^a^	8.85 ± 0.37^a^	8.85 ± 0.37^a^	8.85 ± 0.37^a^	7.57 ± 1.61^ab^	8.42 ± 1.13^a^
	TR-15 (−2b)	4.00 ± 2.51^b^	6.40 ± 1.96^b^	8.71 ± 0.48^a^	8.57 ± 1.13^a^	8.71 ± 0.75^a^	9.00 ± 0.00^a^	5.57 ± 0.97^b^
	NADL (−1a)	1.00 ± 0.00^c^	2.60 ± 2.92^c^	2.28 ± 2.98^b^	9.00 ± 0.00^a^	8.85 ± 0.37^a^	9.00 ± 0.00^a^	8.85 ± 0.37^a^
	Gi-II (−2b)	1.00 ± 0.00^c^	2.40 ± 2.55^c^	2.00 ± 2.23^b^	7.00 ± 2.88^a^	7.42 ± 1.71^a^	6.57 ± 1.27^b^	6.57 ± 1.27^c^

Values are given as mean ± SD

a, b, c, d; Different letters in the same column indicate statistical difference (*p* < 0.05)

Two animals reserved as unvaccinated control (from Group III) remained seronegative during the sampling period

### Efficacy to protect from viremia

Table 2 displays the results of viral antigen detection by ELISA from swabs and serum samples in group I (single dose vaccination), group II (two dose vaccination), and group III (unvaccinated), as well as genome detection by real-time RT-PCR from whole blood samples in group II (two dose vaccination), and III (unvaccinated) day by day after the experimental challenge. Real-time RT-PCR was performed to test the two dose vaccination and unvaccinated group, and the detected Ct values for samples ranged from 14.12 to > 30. Starting on post-challenge (p.c.) day 3 and continuing until day 13, viremia was detected in the unvaccinated animals (group III) on almost all the sampling days (Yeşilbağ et al. 2024). In contrast to the unvaccinated group, there were significantly fewer number of positive results obtained in group II (*p* < 0.05). According to real-time RT-PCR results, all four unvaccinated animals (4/4, 100%) in group III developed viremia for 4 to 10 days, while there were no animals in the vaccinated group II (0/5, 0.0%) detected to be viremic during the study period.

Animals with an OD_450_ value above 0.080 were considered positive in the ELISA test run on each group. Rectal and nasal swab samples taken from animals in all three groups showed negative results for antigen ELISA. Three of 4 animals (75%) in the unvaccinated control group-III, serum samples were found positive in ELISA between days 7th and 11th p.c. There were two cattle detected BVDV antigen positive in the two dose vaccination (group-II, 2/5, 40%), and the three animals in the single dose vaccination (group-I, 3/5, 60%) were positive on days 8–9 p.c (Table 2). As a result, a significant decrease was found after the challenge in group I and group II compared to group III (*p* < 0.05).

**Table 2:** Evaluation of the vaccine efficiency to depression of post-challenge viremia in experimental group-I (n: 5), group-II (n: 5), and group-III (n: 4)

Groups	Animal No	Ag ELISA^a^/real-time RT-PCR ^b^/virus isolation^c^ Results (Days post-challenge)										
		3	4	5	6	7	8	9	10	11	12	13
Group-I (Single dose vaccination)	I-1	-/nd/-	-/nd/-	-/nd/-	-/nd/-	-/nd/-	-/nd/-	-/nd/-	-/nd/-	-/nd/-	-/nd/-	-/nd/-
	I-2	-/nd/-	-/nd/-	-/nd/-	-/nd/-	-/nd/-	+/nd/-	-/nd/-	-/nd/-	-/nd/-	-/nd/-	-/nd/-
	I-3	-/nd/-	-/nd/-	-/nd/-	-/nd/-	-/nd/-	+/nd/-	-/nd/-	-/nd/-	-/nd/-	-/nd/-	-/nd/-
	I-4	-/nd/-	-/nd/-	-/nd/-	-/nd/-	-/nd/-	-/nd/-	-/nd/-	-/nd/-	-/nd/-	-/nd/-	-/nd/-
	I-5	-/nd/-	-/nd/-	-/nd/-	-/nd/-	-/nd/-	+/nd/-	+/nd/-	-/nd/-	-/nd/-	-/nd/-	-/nd/-
Group-II (Two dose vaccination)	II-1	-/-/-	-/nd/-	-/-/-	-/nd/-	-/-/-	+/nd/-	-/-/-	-/nd/-	-/-/-	-/nd/-	-/nd/-
	II-2	-/-/-	-/nd/-	-/-/-	-/nd/-	-/-/-	+/nd/-	-/-/-	-/nd/-	-/-/-	-/nd/-	-/nd/-
	II-3	-/-/-	-/nd/-	-/-/-	-/nd/-	-/-/-	-/nd/-	-/-/-	-/nd/-	-/-/-	-/nd/-	-/nd/-
	II-4	-/-/-	-/nd/-	-/-/-	-/nd/-	-/-/-	-/nd/-	-/-/-	-/nd/-	-/-/-	-/nd/-	-/nd/-
	II-5	-/-/-	-/nd/-	-/-/-	-/nd/-	-/-/-	-/nd/-	-/-/-	-/nd/-	-/-/-	-/nd/-	-/nd/-
Group-III (Unvaccinated)	III-1	-/+/-	-/nd/-	-/+/-	-/nd/-	-/+/-	-/-/-	-/+/-	-/nd/-	-/-/-	-/nd/-	-/-/-
	III-2	-/-/-	-/nd/-	-/+/-	-/nd/-	+/+/-	+/-/-	+/+/-	+/nd/-	+/+/-	-/nd/-	-/-/-
	III-3	-/+/-	-/nd/-	-/+/-	-/nd/-	+/+/-	+/-/-	+/-/-	+/nd/-	-/-/-	-/nd/-	-/-/-
	III-4	-/+/-	-/nd/-	-/-/-	-/nd/-	-/+/-	+/-/-	+/+/-	+/nd/-	-/-/-	-/nd/-	-/-/-

a/b/c: ELISA antigen detection result for serum samples/real-time RT-PCR result for whole blood samples/virus isolation result for rectal and nasal swabs samples of the same animal

The OD_450_ > 0.80 was evaluated positive in ELISA and Ct < 30 is positive in real-time RT-PCR

nd: not done, (+): positive result, (**-)**; negative result

### Depression of change in the body temperature and blood parameters

**Fig. 1:**
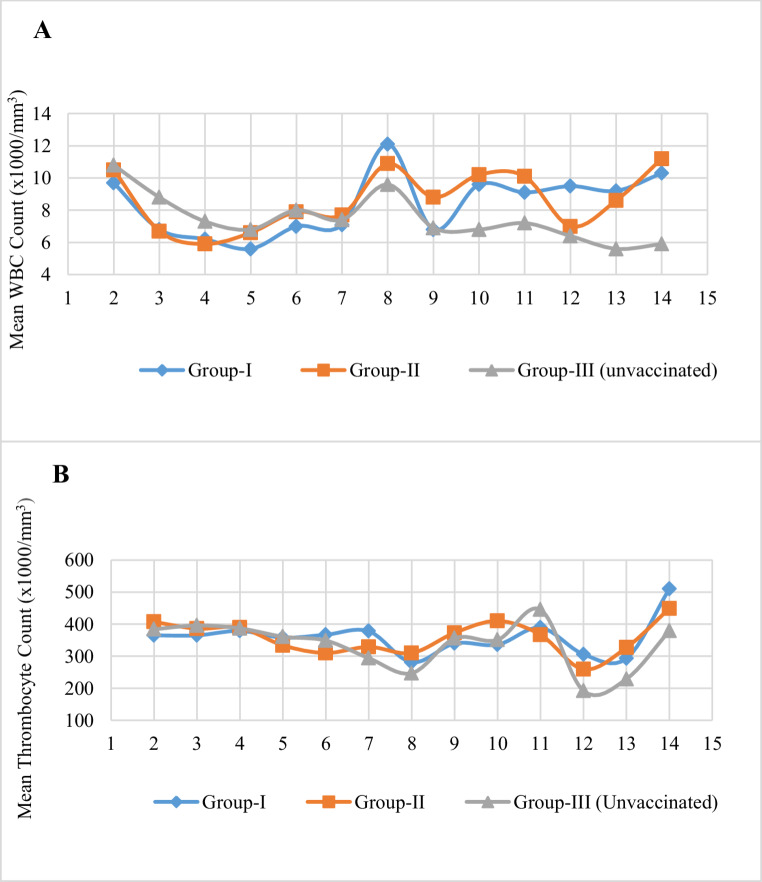
Comparison of the mean WBC (**A**) and thrombocyte (**B**) counts among vaccinated and unvaccinated groups following the experimental challenge

During the first ten days, the groups blood parameters were not statistically different between groups. When the blood test results of the unvaccinated challenge group (group-III) were compared to those of the group-I, the WBC was significantly lower by 39% and 42% on days 13 and 14, respectively (Fig. 1A). On days 10 and 14, however, the WBC rate in group-III was significantly reduced by 33% and 47% in comparison to the group-II. The decrease in platelet count occurred significantly by 37% only on the 12th day between the unvaccinated group and the single dose vaccination group (Fig. 1B, *p* < 0.05). The variations in erythrocyte count across the groups were not statistically significant.

**Fig. 2:**
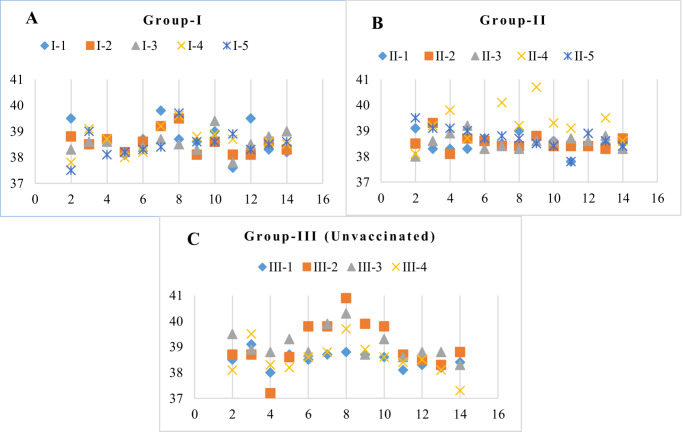
Individual fever scores (°C) of animals after heterologous challenge with BVDV TR-72. (The x axis represents the days post challenge, and the y axis represents the fever degree; Each of the individual animals represented by different symbols)

Individual rectal temperatures of animals in the experimental groups in post-challenge period are shown in Fig. 2. Three out of 5 animals (3/5, 60%) that received a single dose vaccine in Group I experienced high fever (> 39.5 °C) in the post-challenge period; the highest fever recorded was 39.8 °C. A high fever of 39.8 °C to 40.7 °C was noted in only 1 animal (1/5, 20%) in the group-II received the two dose vaccine, and there was a significant difference between the unvaccinated group (group-III) (*p* < 0.05) where high fever (39.7–40.9 °C) was detected in 3 animals (3/4, %75) between the 6th and 10th days p.c. Mean body temperatures exceeded 39 °C for only 1 day (9th p.c.) in group-II, while it was 2 days in group-I (7th, 8th p.c.) and 5 days in group-III (3rd, 7th to 10th p.c.).

### Viral shedding

Following the challenge, no virus was isolated from the tested serum, leukocyte, rectal, and nasal swab samples taken from animals in the single dose and two dose vaccination groups. However, the virus was re-isolated from leukocyte samples (ID: III-2) of an animal from the unvaccinated challenge group (Group-III) on days 5th, 7th, and 8th p.c. Infectious titers were obtained at 3rd blind passage and the titers were 10^6^, 10^5^, and 10^5.75^ TCID_50_/ml, respectively. Meanwhile, the challenge virus was isolated on the 5th day of the leukocyte sample of one animal (ID: III-4) and the 6th day serum sample of one animal (ID: III-2).

### Clinical manifestations

Challenge virus TR-72 (BVDV-1*l*) has been shown to cause serous nasal discharge, mucopurulent nasal discharge, lacrimation, hyperemia, coughing, and malaise in unvaccinated animals (Yeşilbağ et al. 2024). In the present study, the most common sign of serous and mucopurulent nasal discharge was observed in the animals in the experimental groups. Among animals in Group-III (Unvaccinated), hyperemia, ailment, and cough signs intensified on the sixth and ninth days after the challenge (Table 3). The experimental groups who received single dose and two dose vaccines exhibited a significantly reduced range of clinical signs (*p* < 0.05). Furthermore, none of the animals in the study groups exhibited diarrhea. Data on the body temperature findings are already presented above (Fig. 2A-C). No local or systemic allergic reactions or deaths in the animals were recorded after the prime and booster doses of the vaccine.

**Table 3:** Comparison of post-challange clinical findings in vaccinated and unvaccinated groups

Groups	Animal No	Days After Challenges^*^								
		3	4	5	6	7	8	9	10	14
Group-I (Single dose vaccination)	I-1	-	-	-	-	-	-	-	-	-
	I-2	-	-	-	-	-	-	M	-	-
	I-3	-	-	-	-	-	-	-	-	-
	I-4	-	-	-	-	-	-	-	-	-
	I-5	-	-	-	-	M	-	-	-	-
Group-II (Two dose vaccination)	II-1	-	-	-	-	-	-	-	-	-
	II-2	S	-	-	-	-	-	-	-	-
	II-3	-	-	-	-	-	-	S	M	-
	II-4	S	-	-	-	-	-	M	-	-
	II-5	-	-	-	-	-	-	-	-	-
Group-III (Unvaccinated)**	III-1	S, L	M	-	-	-	S	M	-	-
	III-2	-	-	C	H	S	M, C, A	M	-	M
	III-3	-	S	-	H	M, H, C	M, C, A	-	-	-
	III-4	-	-	S	H	-	M, C	S	-	-

*Days when only clinical findings except fever presented

-: No findings in clinical examination

S: Serous nasal discharge, M: Mucopurulent nasal discharge, L: Lacrimation, H: Hyperemia, C: Cough, A: Ailment/malaise

**: Ref. to: (Yeşilbağ et al. 2024)

## Discussion

The global prevalence of BVD infection results in significant financial losses for the livestock industry. BVDV control/elimination programs, although carried out in many countries, are generally not implemented in low- and middle-income countries. Possible insufficiency of vaccines prepared with common vaccinal strains has already been demonstrated (Yeşilbaǧ et al. 2014; Alpay and Yeşi̇lbağ 2019; Evans et al. 2019; Toker et al. 2020). Thus, developing safe and effective vaccines that provide protection against endemic/local strains is crucial. In this study, experimental cattle were immunized with a single dose or two dose vaccination of a novel inactivated vaccine containing local vaccinal BVDV strains, namely TR-21 (BVDV-1*l*), TR-26 (BVDV-1f), and TR-15 (BVDV-2). Then, on one branch, a group of cattle was monthly sampled for follow up the antibody level for seven months, and the second branch of vaccinated cattle was subjected to experimental challenge infection with a BVDV-1*l* (TR-72) strain.

Currently, the killed vaccines are the widely used vaccine technology for cattle vaccines including BVDV (Hamers et al. 2003; Makoschey et al. 2007). Despite various adjuvant systems could be preferred, the oil-based adjuvants share advantages as work by retaining the vaccine antigen and releasing it over an extended period, which boosts the immune response. The priority of Montanide^®^ oil based adjuvant was assessed in an experimental model evaluating the adverse effects and efficacy of five distinct oil-based adjuvants (Leenaars et al. 1998). Also, the Montanide^®^ series has different effects on inducing immune response, with ISA 206 eliciting a protective immunological response faster than ISA 50 (Ibrahim et al. 2015). Similarly, in vaccine studies using the ISA 206 adjuvant, it produce a considerable and highly immunogenic effect in vaccinated calves for a long period of 9–12 months (I.EL-Hawary and Mostafa 2017; El-Bagoury et al. 2020). The power of this adjuvant including compatibility with the used viral antigens was also experimented during our preclinical studies in laboratory animals (Kadiroğlu and Yeşilbağ 2025). Hence, Montanide ISA 206 was selected as the suitable adjuvant for the experimented vaccine. Evaluation of vaccine potency has shown that the ISA 206 adjuvant vaccination maintains protective neutralizing antibody (nAb) levels against both homologous and heterologous strains for more than seven months after vaccination (Table 1). Furthermore, it was also practiced to be finely applicable to mix with the selected antigens and apply to the target animals.

To prevent clinical signs of BVDV infection and viremia, the nAb titer is suggested to be 2.4 log_2_ and above (Bolin and Ridpath 1990). In the present study, nAb titers against homologous strains began to develop on day 21st and peaked till the days 81st and 171st after immunization. For the heterologous strains (NADL and Gi-II), despite the titers began to rise on day 21st, the main increase was on day 111st for the single dose vaccination group (Group-I) and on day 51st for the two dose vaccination group (Group-II). Those data represent the increasing humoral immunity for a long period. In single dose vaccination group first nAb titer values above protective antibody titer were detected at 21–51 days against strains included in the vaccine and obtained against both reference strains between days 111 and 141 after immunization. However, in the two dose vaccination vaccine group, it was observed that the nAb titer responses increased on the 21st day against the strains included in the vaccine and on the 51st day against the reference strains. Even though a protective level of nAb development was found in both groups, the earlier onset of the suggested antibody response in the two dose vaccination group is crucial for avoiding infection (Makoschey et al. 2007). Our data also refers requirement of a booster dose of the studied vaccine formulation for better results for gaining nAb response against heterologous strains. Although our vaccine formula does not contain the BVDV-1a strain, a protective antibody response (at day 111) was obtained against the BVDV-1a subtype which is included in almost all BVDV vaccines cause of including BVDV-1 reference strain. In the vaccine group that received a booster dose, this response was found to be as early as the 51stday. Experimental inactivated BVDV vaccine studies have shown that genotype BVDV-1 may protect BVDV-2 (Beer et al. 2000; Makoschey et al. 2001; Hamers et al. 2003). However, examination of field conditions revealed that vaccinated animals can be infected and there were serological differences between strains even within the same genotype (Fulton et al. 1997; Alpay and Yeşilbağ 2015). For this reason, it was deemed appropriate to include the BVDV-2 subgroup also in our vaccine formulation. In the present study, antibody titers against the BVDV-2 Gi-II strain were relatively lower than those against other strains; yet, the increasing titer eventually increased the protective level in both groups and reached a maximum value of three times of suggested protective nAb (Table 1). In studies on immunity against BVDV strains, it has been shown that there are differences in antibody titers obtained despite being of the same genotype. This may be due to differences in immunodominant epitopes or antigen-specific T cell responses between strains. The protective immune response may include both “general” and “targeted species/genotype/strain” specific antibodies and cell-mediated immunity responses (Falkenberg et al. 2021). The nAbs developed for BVDV-2 virus strains (TR-15 and Gi-II) were nearly 2 times higher in the single dose vaccination and 2 times higher than the suggested protective antibody response in the two dose vaccination group, also lasting ≥ 7 months. On that point, two dose vaccination looks to be leading to a protective nAb level at the 30th day after the booster vaccination, which looks to be 60 days earlier comparing to single dose vaccination.

Data on different clinical findings in the TR-72 experimental infection have been determined previously (Yeşilbağ et al. 2024). In the present study, only serous and mucopurulent nasal discharge was detected in animals that received single dose and two dose vaccination during the challenge. The observed findings were significantly less common than in the unvaccinated group, both in terms of the type of clinical findings and the frequency of occurrence in the animals. In addition, the number of hyperthermic animals was less in both of the vaccinated groups (Groups–I and–II) than in the unvaccinated group (Group-III), and significantly lower temperatures were in the two dose vaccination group, while the highest fever (40.9 °C) post-challenge was in the unvaccinated animals (*p* < 0.05, Fig. 2A-C). Our data demonstrated that single dose vaccination and two dose vaccination administration of the studied vaccine significantly reduced the clinical signs (Table 3, *p* < 0.05). However, it is not possible to attribute the severity and frequency of the current clinical findings to the challenge virus because the presence of other infectious agents was not assessed throughout the challenge infection. On the other hand, the days on which fever peaks, clinical signs, and the presence of the virus in the blood of unvaccinated animals are convenient at the point of pathogenesis with studies of experimental BVDV infection (Makoschey et al. 2001; Walz et al. 2001; Liebler-Tenorio et al. 2002; Hamers et al. 2003; Ridpath et al. 2007).

The BVD virus induces hematological changes that result in transient thrombocytopenia and leukopenia by mechanisms such as lesions in the lymphoid tissue, bone marrow necrosis, megakaryocyte hyperplasia, direct attack on platelets or immune-mediated platelet destruction (Scruggs et al. 1995). Thus, changes in blood parameters including WBC, platelets, and less frequent erythrocytes have been the main focus of many BVDV experimental infection research (Makoschey et al. 2001; Hamers et al. 2003; Xue et al. 2010; Glotov et al. 2016; He et al. 2020). In our study, although there were significant decreases in WBC and platelet counts in unvaccinated animals during challenge infection (*p* < 0.05), all the animals in the vaccinated experimental groups (groups I and II) were within the reference values, with counts ranging from 5 to 12 (x1000/mm^3^) and 253–510 (x1000/mm^3^), respectively. On the other hand, a measurable decrease in WBC was detected between days 2 and 5. Since no statistically significant difference was found between the experimental groups, this situation is not thought to be due to the experiment. However, it is not possible to explain the reason for this decrease with our current experimental setup. In this study, there was no significant change in the RBC counts of cattle in all the groups after challenge infection. Studies have shown that RBC responses can vary such as decrease, increase, or no change (Scruggs et al. 1995; Hamers et al. 2003; Kocatürk et al. 2010). These differences were attributed to bone marrow hypoplasia and necrosis, decreased erythropoiesis, hemolysis, or activated bone marrow, but the exact pathogenesis remains unclear. In short, inconsistency in changes in erythrocyte count has been associated with the virus in response to different virulent BVDV isolates and the severity of BVDV infection.

The two dose vaccination significantly depressed the development of the viremia phase as detected by virus isolation study, real-time RT-PCR, and ELISA (*p* < 0.05, Table 2). This result, which is consistent with reduced clinical findings in vaccinated animals (group-I, and–II), is in line with previous studies (Table 3) (Hamers et al. 2003). The quantity of virus shedding in the field conditions has a significant impact on both the rate of virus transmission and the severity of an outbreak. Nasal and rectal swab samples in which the virus was not isolated in the single and two dose vaccination groups were confirmed by BVDV Ag ELISA. The infective virus was isolated only from leukocyte and serum samples of animals in the unvaccinated group during their third passage in cell culture. Therefore, virus isolation and PCR results obtained from blood samples reveal that the tested vaccine suppresses the development of viremia (Table 2).

In conclusion, our data show that reduced clinical findings and hematological changes were detected in the vaccinated animals which also prevented from development of viremia. This study showed promise since the vaccine formulation prepared using common or endemic local strains protected the vaccine against both homologous and heterologous strains. Although it is not included in this vaccine, it has enabled the development of high protective responses not only against the BVDV-1 reference strain, but also against BVDV-1 subgroups that are quite or most common in some countries, and the BVDV-2 reference strain. Montanide ISA 206 oil-based adjuvant, a remarkably safe, well-tolerated adjuvant used in animal vaccines, without causing local or systemic allergic reactions was found compatible with the selected vaccine formulation. The inactivated trivalent vaccine is a potentially safe and useful tool in the fight against the severe impacts of BVDV on cattle. The vaccination would undoubtedly help livestock farmers financially by enabling the containment of epidemics.

## Data Availability

No datasets were generated or analysed during the current study.
